# Epigenetic mechanisms of endothelial dysfunction in type 2 diabetes

**DOI:** 10.1186/s13148-015-0090-4

**Published:** 2015-05-23

**Authors:** Francesco Prattichizzo, Angelica Giuliani, Artan Ceka, Maria Rita Rippo, Anna Rita Bonfigli, Roberto Testa, Antonio Domenico Procopio, Fabiola Olivieri

**Affiliations:** Department of Clinical and Molecular Sciences, DISCLIMO, Università Politecnica delle Marche, Via Tronto 10/A, 60020 Ancona, Italy; Scientific Direction, National Institute INRCA-IRCCS, Ancona, Italy; Center of Clinical Pathology and Innovative Therapy, National Institute INRCA-IRCCS, Ancona, Italy; Experimental Models in Clinical Pathology, National Institute INRCA-IRCCS, Ancona, Italy

**Keywords:** Epigenetic markers of T2DM, microRNA, Metabolic memory

## Abstract

The development of type-2 diabetes mellitus (T2DM) and its complications is largely due to the complex interaction between genetic factors and environmental influences, mainly dietary habits and lifestyle, which can either accelerate or slow down disease progression. Recent findings suggest the potential involvement of epigenetic mechanisms as a crucial interface between the effects of genetic predisposition and environmental factors. The common denominator of environmental factors promoting T2DM development and progression is that they trigger an inflammatory response, promoting inflammation-mediated insulin resistance and endothelial dysfunction. Proinflammatory stimuli, including hyperglycemia, oxidative stress, and other inflammatory mediators, can affect epigenetic mechanisms, altering the expression of specific genes in target cells without changes in underlying DNA sequences. DNA methylation and post-translational histone modifications (PTHMs) are the most extensively investigated epigenetic mechanisms. Over the past few years, non-coding RNA, including microRNAs (miRNAs), have also emerged as key players in gene expression modulation. MiRNAs can be actively released or shed by cells in the bloodstream and taken up in active form by receiving cells, acting as efficient systemic communication tools. The miRNAs involved in modulation of inflammatory pathways (inflammamiRs), such as miR-146a, and those highly expressed in endothelial lineages and hematopoietic progenitor cells (angiomiRs), such as miR-126, are the most extensively studied circulating miRNAs in T2DM. However, data on circulating miRNA signatures associated with specific diabetic complications are still lacking. Since immune cells and endothelial cells are primarily involved in the vascular complications of T2DM, their relative contribution to circulating miRNA signatures needs to be elucidated. An integrated approach encompassing different epigenetic mechanisms would have the potential to provide new mechanistic insights into the genesis of diabetes and its severe vascular complications and identify a panel of epigenetic markers with diagnostic/prognostic and therapeutic relevance.

## Review

### Introduction

Type-2 diabetes mellitus (T2DM) is a chronic multifactorial metabolic disease caused by a complex interaction between environmental and genetic factors [1]. T2DM is a source of disability and morbidity related mainly to vascular complications which underlie the development of retinopathy, nephropathy, neuropathy, ischemic heart disease, and peripheral vasculopathy [2, 3]. Endothelial dysfunction (ED) is the key etiological factor that induces moderate to severe vascular complications and has been proposed as a key therapeutic target in T2DM patients [4, 5]. Early exposure to hyperglycemia can involve disease progression and late complications, perpetuating ED despite the achievement of improved glycemic control; the phenomenon has been called “metabolic memory” [6]. A variety of mechanisms contribute to metabolic memory, including increased production of advanced glycation end-products (AGEs), AGE-receptor (RAGE) overexpression, increased anion superoxide formation, mitochondrial protein glycation, mitochondrial (mt)DNA damage, protein kinase C (PKC) activation, and polyol pathway and hexosamine flux alterations [6]. As a consequence of metabolic memory, the risk of diabetic complications escalates over time despite combined treatment with glucose-lowering drugs, anti-hypertensives, and anti-inflammatory agents [7]. Notably, the concept of metabolic memory refers mainly to vascular stress and damage persisting after glucose normalization; recent evidence shows that oscillating glucose may be more harmful than hyperglycemia itself [8].

A number of genes involved in susceptibility to T2DM and its complications have been identified by linkage studies, candidate gene association studies, genome-wide association studies, and meta analyses in diverse ethnic groups [9]. At present, however, genetic testing cannot accurately predict the clinical risk of T2DM and/or its complications, suggesting that the disease is not entirely accounted for by genetic predisposition [10, 11].

Recent data suggest that epigenetic mechanisms may be a crucial interface between the effects of genetic predisposition and environmental factors [12, 13]. Transient hyperglycemia may mediate persistent gene-activating events underlying metabolic memory and sustain ED, despite the achievement of a good glycemic control [14].

DNA methylation and post-translational histone modifications (PTHMs) are the most extensively investigated epigenetic mechanisms involved in metabolic memory. PTHMs and DNA methylation can become irreversible over time, explaining the long-lasting detrimental effects of metabolic memory, which induce T2DM vascular complications even after improvement of glycemic control.

Epigenetic mechanisms may thus at least partly explain the link between factors acting during fetal life and the later risk of developing T2DM [15]. In mammals, the DNA methylation pattern is largely established during embryo development [16, 17]. Once formed, DNA methylation patterns must be maintained during cell division to preserve cell identity, even though some changes are observed during chronological aging [18]. Therefore, factors acting during prenatal life capable of inducing epigenetic modifications in different tissues and organs, such as malnutrition or stress, may have a long-term effect by increasing the risk of T2DM and coronary heart disease in later life [19]. It is therefore conceivable that epigenetic modifications in fetal life set a range of parameters—such as insulin sensitivity and secretion, hepatic glucose production, and synthesis and release of hormones involved in glucose and insulin metabolism—affecting the risk of T2DM development in adult life.

Additional epigenetic mechanisms have recently been identified. Non-coding RNA, including microRNAs (miRNAs), have emerged as key factors in gene-expression modulation and could play a role in the modulation of metabolic memory. More than 2000 human miRNAs have been identified, making them one of the most abundant classes of epigenetic regulatory molecules [20]. MiRNAs were previously thought to act mainly as negative regulators of gene expression by binding to 3-UTR regions of their target protein-coding mRNAs in a sequence-dependent manner [21]. However, a growing body of evidence supports the notion that miRNAs are not only post-transcriptional regulators of gene expression. Indeed, they can indirectly modulate the methylation of promoter or coding sequences by targeting enzymes involved in methylation [22] and can directly repress or stimulate target-gene transcription by direct binding to promoter regions, a phenomenon that has been designated RNA activation (RNAa) [23]. Interestingly, a class of 24 nucleotide-long plant miRNAs (lmiRNAs) are capable of recruiting methyltrasferases, thus directly modulating DNA methylation [24]. lmiRNAs bound to AGO4 proteins interact with the nascent transcripts transcribed from their own loci or target genes, thereby recruiting *de novo* cytosine methyltransferase DRM2 to methylate adjacent DNA [24]. Furthermore, miRNA genes are extensively regulated at the level of promoter methylation, transcription, and processing [23].

Importantly, miRNAs can be actively released or shed by cells in the bloodstream and taken up in active form by receiving cells, acting as efficient systemic communication tools. Thanks to their easy detection in serum and plasma, stability under a variety of storage conditions, and ability to be measured by sensitive, specific assays (e.g., quantitative RT-PCR), they are emerging as minimally invasive, inexpensive biomarkers of complex processes like age-related diseases including T2DM and its complications [25].

MiRNAs are being demonstrated to be functional biomarkers capable of co-ordinating multiple pathways and modulate virtually all cellular responses to environmental stimuli according to each individual’s genetic makeup. Factors associated with diabetic complications, such as hyperglycemia, oxidative stress, and inflammation, can induce deregulation of epigenetic mechanisms, resulting in modification of circulating miRNA profiles. Consequently, the expression of specific genes in target cells, especially in endothelial and vascular smooth muscle, can be changed without inducing modification in the underlying DNA sequence [14].

Circulating miRNAs are thus expected to be informative, easily accessible, and cost-effective candidate biomarkers of the age-related disease development and progression, enabling assessment of the health status of individuals both at the level of specific tissues/organs and at the systemic level [25]. Only an integrated approach that considers different epigenetic mechanisms as a whole may have the potential to provide new mechanistic insights into the genesis of the vascular complications of diabetes and identify a panel of epigenetic markers, “epi-markers”, with diagnostic/prognostic relevance. Circulating miRNAs as well as DNA/histone methylation/acetylation can be considered as candidate “epi-markers”. Innovative therapeutic strategies for the chronic complications of T2DM should focus on deleting metabolic memory by targeting enzymes involved in methylation/acetylation of DNA and histones and by modulating miRNA expression levels.

Here, we review the latest data on epigenetic mechanisms in relation to their ability to modulate T2DM progression, discuss the possibility of using epi-markers as diagnostic and prognostic markers of T2DM and its complications and provide an up-to-date perspective on the potential of using circulating miRNAs as biomarkers of T2DM and its complications.

#### Epigenetic mechanisms involved in the persistence of metabolic memory and in the development of T2DM complications

Aging is associated with a chronic systemic inflammatory state, named inflammaging, which greatly contributes to diabetes onset and progression [26, 27]. The similarity of the risk factors for cardiovascular disease (CVD) and diabetes has generated the hypothesis of a shared inflammatory basis [28]. The repeated stimulation of innate immune response over time and the accumulation of senescent cells during aging are the main contributors to inflammaging. Senescent cells can acquire a phenotype that is closely related to the senescence phenotype, named senescence-associated secretory phenotype (SASP) [29]. Even though senescence does not necessarily entail SASP acquisition, as demonstrated by the fact that ectopic expression of the cyclin-dependent kinase inhibitor p16INK4a induces senescence in the absence of a functional SASP, the phenotype is commonly associated with both replicative and induced senescence [30, 31].

Albeit in growth arrest, senescent cells remain metabolically active, secreting several different bioactive molecules, i.e., cytokines, growth factors, metalloproteinases, and other metabolites that contribute to induce and maintain a proinflammatory microenvironment [32]. The inflammatory phenotype, which is typical of the cells involved in immune responses, including endothelial cells (ECs), triggers the activation of nuclear factor kappa B (NF-kB)-dependent signaling, which induces transcription of a number of genes involved in the modulation of inflammatory response, including adhesion molecules, such as VCAM-1, and cytokines, such as interleukin (IL)-1, IL- 6, and TNFα [33]. Notably, these genes are chronically activated in cells from diabetic patients [34, 35]. Even though the mechanisms involved in the maintenance of inflammaging are not completely clear, emerging evidence suggests that epigenetic mechanisms could be involved, contributing to the development of diabetes and its vascular complications [36]. Since co-ordinated changes involved in chromatin remodeling are detected days after exposure to hyperglycemia even after restoration of normoglycemic conditions, such epigenetic modifications may be involved in maintaining metabolic memory. A number of studies are currently exploring epigenetic mechanisms in the expression of proinflammatory genes. Notably, modulators of methylation status, histone-modifying enzymes, and specific miRNAs may provide a novel strategy to prevent T2DM and its complications.

Recent data have disclosed glucose-mediated changes in the transcription and activation of NF-kB in ECs and peripheral blood cells exposed to transient hyperglycemia, or obtained from diabetic patients. In particular, some epigenetic alterations have been observed in the NF-kB promoter region, leading to increased expression of NF-kB subunit p65, such as: 1) recruitment of the histone methyltransferase Set7 and increased monomethylation of H3K4 (lysine 4 of histone 3) [37, 38]; 2) increased recruitment of the histone demethylase LSD1 and reduced H3K9 methylation; and 3) histone acetyltransferase (HAT)-mediated histone H3K9 hyperacetylation. Moreover, CREB-binding protein (CBP) and CBP-associated factor (P/CAF) are NF-kB coactivators possessing intrinsic HAT activity and are involved in NF-kB activation [39]. Reversible acetylation of NF-kB subunits induces an intranuclear molecular switch controlling the duration of NF-kB transcriptional response [40]. Therefore, histone acetylation/deacetylation through HAT could constitute an innovative therapeutic strategy to counteract hyperglycemia-induced inflammation [41]. Of interest, in human aortic endothelial cells (HAECs), Set7 silencing prevented H3K4 monomethylation and abolished NF-kB-dependent oxidant and inflammatory signaling, suggesting that Set7 could be targeted to erase metabolic memory and avoid vascular complications [42, 43].

Increasing data have disclosed glucose-mediated changes in the transcription of other genes expressed in ECs and involved in modulation of inflammation. In particular, rapid histone H3K9/K14 hyperacetylation is associated with enhanced expression of HMOX1 (heme oxygenase (decycling) 1), IL-8, and matrix metalloproteinase 10 (MMP-10) in HAECs exposed to hyperglycemia [44].

Histone H3K9 acetylation has been detected in monocytes from diabetic patients as having TNFα overexpression and enhanced activation of cyclo-oxygenase (COX)-2, which have an important role in the induction and maintenance of inflammatory processes [41]. In conclusion, H3K9 hyperacetylation of a variety of gene promoters (e.g., HMOX1, IL-8, MMP-10, TNFα, and COX-2) is a common feature of vascular and immune cells in diabetic patients [41, 44].

Interestingly, epigenetic mechanisms are closely interconnected, and specific miRNAs can modulate epigenetic signatures through different mechanisms, including targeting of methyltransferases [45]. In myocardial vascular smooth muscle cells from diabetic mice, miR-125b elevation induced repression of the methyltransferase Suv39h1, which in turn was associated with reduced H3K9me3 expression in the promoter region of inflammatory genes such as MCP-1 and IL-6, resulting in increased expression of the cytokines [46]. The methylation status of the miR-125b gene can be controlled by DNA methyltransferase 1 (DNMT1) [47]. DNMT1 overexpression may result from activation by reactive oxygen species and hyperglycemia in different cell models [47, 48]. However, a direct link between DNMT1 and miR-125b in diabetes remains to be demonstrated.

Genome-wide analysis of lysine-methylation changes in THP-1 cells, a human monocyte cell line, exposed to hyperglycemia and in monocytes from diabetic patients has documented differential changes in H3K9 dimethylation at PTEN-coding and IL-1A promoter regions [49]. However, it is unclear whether the diabetic environment triggers the same epigenetic modifications in ECs.

The above epigenetic studies have often described an association of different PTHMs with mediators that are involved in the vascular complications of diabetes. Chromatin immunoprecipitation (ChIP) combined with DNA array analysis (ChIP-on-chip) has been used for years to acquire genome-wide information on histone modifications [50]. Wider high-resolution maps of DNA methylation (methylome), not focusing on one particular modification/residue, can now be obtained using next generation sequencing devices [51, 52], and could provide insights into disease pathogenesis and identify biomarkers or potential drug targets.

Genome-wide methylated DNA immunoprecipitation sequencing (MeDIP-seq) in whole-blood-derived DNA from 27 monozygotic twin pairs has disclosed a large role for MALT1 (mucosa-associated lymphoid tissue lymphoma translocation protein 1) gene in T2DM incidence [53]. MALT1 is involved in NF-kB activation through the formation of the Carma1-Bcl10-Malt1 (CBM) complex, which is essential for activation of I kappa B kinase (IKK) [54]. A similar approach to the complications of diabetes could provide a signature with strong predictive potential for patients at risk of its vascular complications.

Other epigenetic mechanisms have also been described in association with T2DM, such as reduced Long Interspersed Nucleotide Element 1 (LINE-1) DNA methylation [55]. DNA methylation measured in LINE-1 sequences has been considered a surrogate marker for global genome methylation. Low methylation in LINE-1-repetitive elements has been associated with chromosomal instability as well as inflammatory process [56]. Notably, lower LINE-1 DNA methylation levels in peripheral blood cells were associated with a higher risk of T2DM, independently from other classic risk factors, highlighting the potential role for these epigenetic biomarkers as predictors of T2DM risk or other related metabolic disorders [55].

#### Circulating miRNAs as biomarkers of T2DM and its complications

The common denominator of environmental factors identified as risk factors for the most common age-related diseases, including T2DM, is that they trigger an inflammatory response [57]. Notably, the chronic low-grade systemic inflammation occurring during physiological aging accelerates the development of the age-related diseases, including T2DM [58]. Proinflammatory cytokines receptors, NOD-like Receptors (NLRs) and Toll-like receptor (TLR) pathways play a significant role in the pathogenesis of inflammaging and inflammation-mediated insulin resistance [59–61]. TLR ligands, such as free fatty acids and lipid derivatives from adipocytes and skeletal muscle, activate the TLR pathway inducing NF-kB activation, thus promoting inflammation-mediated insulin resistance and inflammation-mediated ED [61]. TLRs pathway genes are modulated by different epigenetic mechanisms, including miRNAs, the shortest non-coding RNAs involved in gene expression modulation. MiRNAs are expressed by all living cells and can be secreted or released by cells within small membranous vesicles (e.g., exosomes, microparticles, and apoptotic bodies) or packaged in high-density lipoproteins (HDLs), or RNA-binding proteins (e.g., Argonaute) [62–65]. MiRNAs circulate in the bloodstream in a remarkably stable form [66, 67]. Even though in most instances where the origin of circulating miRNAs is unclear, they have been extensively studied as possible biomarkers for a wide range of human diseases, such as cancers, CVD, and immunological, neurodegenerative, and metabolic diseases, including diabetes [68].

**Fig. 1:**
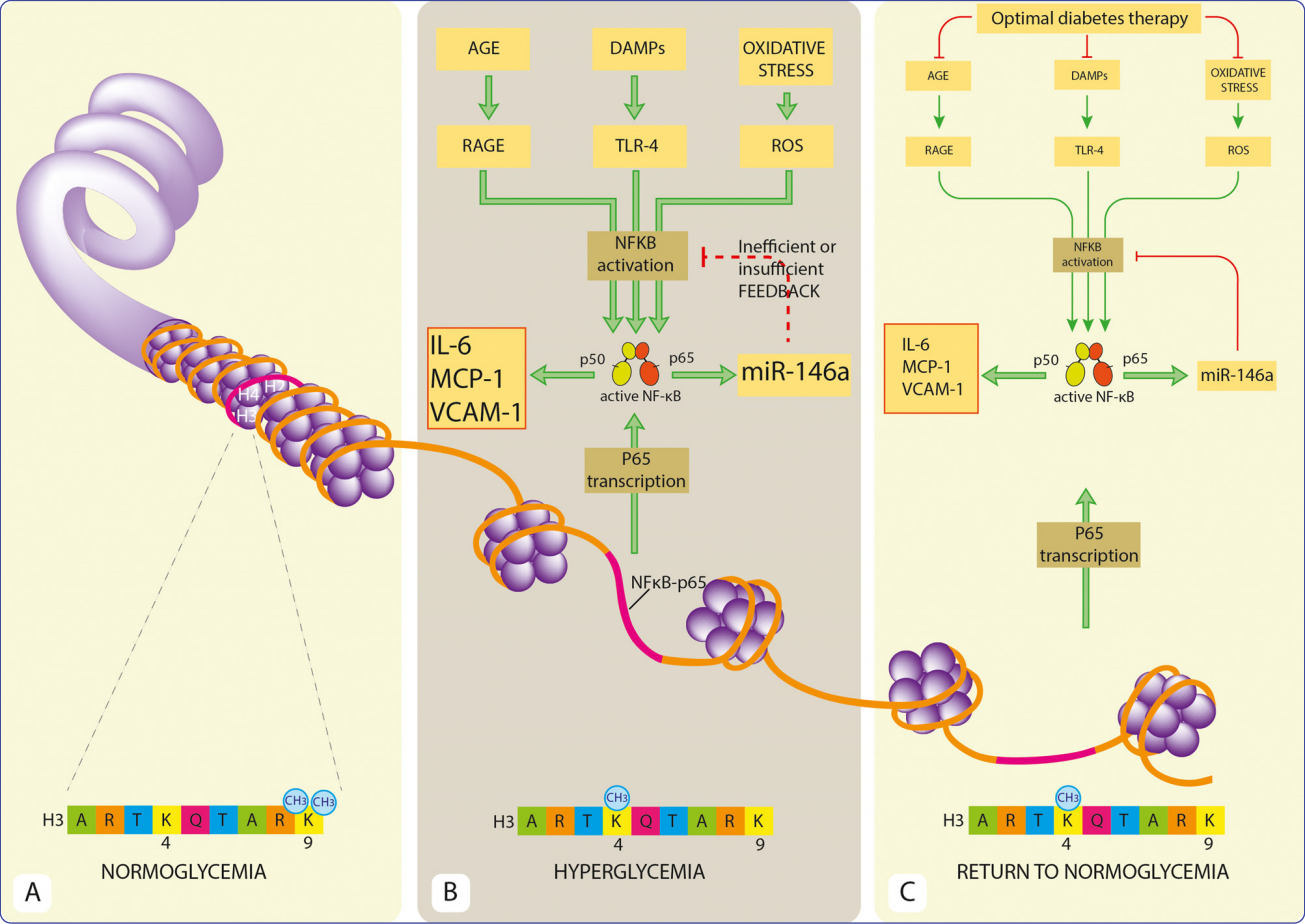
Glycemia-associated epigenetic mechanisms involved in NF-kB proinflammatory activity. Panels **a**–**b**. Cells exposed to high glucose exhibit upregulated expression of the NF-kB subunit of p65 gene, monomethylation of histone 3 at lysine 4 (H3K4), and demethylation of H3K9 in the p65 promoter region. Moreover, hyperglycemia induces NF-kB transcription factor activation through stimulation of upstream pathways, increasing the synthesis of inflammatory mediators (IL-6, VCAM-1, MCP-1) and the expression of anti-inflammatory microRNAs, e.g., miR-146a. Under persistent hyperglycemic conditions miR-146a cannot restrain the effect of upstream proinflammatory stimuli on NF-kB activation. Panel **c** shows that anti-diabetic agents can reduce proinflammatory stimuli on the NF-Kb pathway, restraining p65 activation and miR-146a expression but does not alter H3K4 monomethylation and H3K9 demethylation in the p65 promoter region. The phenomenon may explain why the cardiovascular complications of diabetes progress even in presence of optimal glycemic control. *AGE*, advanced glycation end-products; *DAMP*, damage-associated molecular patterns; *IL*-*6*, interleukin-6; *MCP*-*1*, monocyte chemoattractant protein-1; *RAGE*, receptor for advanced glycation end-products; *VCAM*-*1*, vascular cell adhesion molecule-1; *TLR*-*4*, toll-like receptor 4

We recently defined a number of miRNAs involved in the modulation of TLR pathways as “inflammamiRs” [25]. Notably, the majority of inflammamiRs have been detected not only in tissue but also in plasma and other body fluids, suggesting that they could be involved in the cross-talk between tissues and organs that characterizes systemic inflammation. MiR-146a is the best characterized inflammamiR; it is involved in restraining inflammation, switching off the acute inflammation after removal of the harmful stimulus [69]. Under chronic stimulation, it is overexpressed in different cell types, including ECs and white blood cells [70, 71]. Altered, either increased or decreased, miR-146 expression has been associated with several diseases, including diabetes [72–78]. MiR-146a is down-regulated in peripheral blood mononuclear cells (PBMCs) of T2D patients [72, 73], whereas in plasma of T2DM patients, both reduced and increased miR-146a expression have been described [74–76]. Since different cell types can contribute to circulating miRNA levels, and since hyperglycemia is expected to have different effects on different cell types, contributing to the circulating miRNA pool, miRNA expression is necessarily different in PBMCs and serum. Notably, increased levels of circulating miR-146a have been reported only in newly diagnosed, treatment-naive T2DM patients. MiR-146a expression has also been assessed in animal models of diabetes, like diabetic mice. A positive correlation has been described between miR-146a levels, NF-kB activation, and levels of inflammatory mediators [77, 78]. Moreover, miR-146b-3p, of the miR-146 family, is altered in the vitreous of diabetic patients with retinopathy [79]. The epigenetic modulation of NF-kB transcription/activation in immune cells or ECs is an example of how different epigenetic mechanisms can synergistically interact to maintain a specific phenotype, i.e., metabolic memory, over time (Fig. 1). It may be hypothesized that in the presence of proinflammatory stimulation, induced by chronic hyperglycemia, miR-146a fails to restrain the increased levels of inflammatory markers (Fig. 1a–b). Despite the eventual achievement of glycemic control, the persistence of epigenetic modifications induced by the transient hyperglycemia, such as H3K9 monomethylation in the p65 promoter region, induces an increased NF-kB transcription, which in turn, maintains a weakly increased expression of the proinflammatory mediators (Fig. 1c).

This strongly suggests that a consistent reduction of NF-kB activity in immune cells and ECs of diabetic patients requires a reduction in both NF-kB transcription and activation through modulation of DNA methylation, histone modifications, and miRNAs expression.

Genetic variability at miR-146a loci seems to be unrelated to T2DM incidence; inconclusive data were reported on the association between single nucleotide polymorphism (SNP) rs2910164 of miR-146a gene and T2DM incidence in a large cohort of Chinese Han subjects [80, 81]. However, miR-146a/rs2910164 and fasting glucose have been reported to exert significant combined effects on ischemic stroke susceptibility [81].

Other miRNAs involved in inflammatory pathway modulation have been associated with diabetes. In a recent, important meta-analysis, 40 circulating miRNAs, including miR-126, miR-375, miR-29a, miR-34a, miR-103, miR-107, miR-132, miR-142-3p, and miR-144 are significantly deregulated in T2DM [82]. MiR-126 is the most extensively studied circulating miRNA in T2DM. It promotes vascular regeneration by functioning as an angiomiR and by modulating the mobilization of hematopoietic stem/progenitor cells. A number of reports have shown its down-regulation in diabetic patients [83–85]. We previously reported that circulating levels of miR-126 increase during physiological aging and that the phenomenon is paralleled by increased miR-126 synthesis and release in ECs undergoing senescence in vitro [84]. When we compared diabetic patients to age-matched healthy controls, we found reduced circulating miR-126 levels in T2DM patients, especially the oldest ones [84], suggesting that circulating miR-126 behaves differently in aging and diabetes. This apparent paradox can partially be explained by hypothesizing that the aging/senescence-associated miR-126 up-regulation is a senescence-associated compensatory mechanism that is blunted when ECs are exposed to high glucose levels; a phenomenon that probably occurs in T2DM patients.

No clear data are available on circulating miR-126 in relation to diabetes complications. Increased levels of circulating miR-126 have been reported in patients with coronary artery disease (CAD) [86], and in a recent prospective study, miR-126 levels were found to be positively associated with incident myocardial infarction [87]. However, significantly reduced miR-126 levels were found in circulating microparticles from CAD patients with T2DM [88]. Overall, these data suggest that different circulating miR-126 levels may be found in diabetic patients with cardiovascular complications compared with those with other diabetic complications.

MiR-21 is an extensively studied miRNA in tumor research, since it has been identified as an oncomiR. Notably, our group first identified it as an inflammamiR [89]. MiR-21 down-regulation has been described in serum and in endothelial progenitor cells of diabetic patients [83]. Regarding diabetic complications, increased miR-21 levels have been reported in diabetic patients with proliferative diabetic retinopathy [90]. Moreover, a tissue-specific increase in miR-21 has been reported in different hyperglycemic environments [91, 92]. Interestingly, miR-21 up-regulation in glomerular tissue seems to be a compensatory mechanism to counteract kidney failure in diabetic patients [93].

Analysis of plasma miR-375 expression showed that it was down-regulated in patients with impaired glucose tolerance (IGT) compared with those with normal glucose tolerance (NGT), whereas patients with frank T2DM showed an opposite trend, with significantly increased circulating miR-375 compared with healthy subjects [94]. Analysis of the methylation status of the miR-375 gene in the same cohort of patients disclosed that it was increased in IGT patients compared both with NGT and T2DM patients. This suggests the possibility of different epigenetic modifications in relation to disease stage, and differential expression of circulating miRNAs is found in diabetic patients with different degrees of glycemic control [84, 94].

Overall, even though some circulating miRNAs appear to be candidate biomarkers for T2DM (*i.e*. miR-126), only few data are available on specific circulating miRNAs as biomarkers of diabetic complications [95]. Since several tissues can be involved in diabetic complications, it is conceivable that they provide a different relative contribution to circulating miRNA signatures.

A list of miRNAs differentially expressed in plasma, serum, or whole blood from T2DM patients compared with healthy subjects is reported in Table 1, and a list of miRNAs differentially expressed in tissues from patients with the complications of diabetes or in human cell lines exposed to hyperglycemia is reported in Table 2.

**Table 1:** Circulating miRs differentially expressed in T2DM patients and healthy subjects

Circulating miRs	Expression in T2DM patients *vs*. CTR	Sample type	Refs	miR classification
let-7a	Down	Plasma	[96]	O
let-7f	Down	Plasma	[96]	O
let-7i	Down	serum	[97]	O
miR-124a	Up	serum	[98]	I
miR-125b	Down	Plasma	[85]	O, I
miR-126	Down	Plasma	[85]	A, I, O
	Down	Plasma	[99]	
	Down	Plasma	[83]	
miR-130b	Down	Plasma	[85]	O
miR-140-5p	Up	Plasma	[85]	
miR-142-3p	Up	Plasma	[85]	I, O
miR-144	Up	Peripheral blood	[74]	O, I
miR-146a	Up	Serum	[98]	I, A, O
	Up	Plasma	[76]	
	Down	Serum	[97]	
	Down	Peripheral blood	[74]	
	Down	Serum	[75]	
miR-150	Up	Peripheral blood	[74]	I, A, O
miR-15a	Down	Plasma	[83]	O
miR-182	Down	Peripheral blood	[74]	O
miR-186	Down	Serum	[97]	O
miR-191	Down	Serum	[97]	O
	Down	Plasma	[83]	
miR-192	Down	Plasma	[85]	O
	Down	Serum	[97]	
	Up	Peripheral blood	[74]	
miR-195	Down	Plasma	[85]	A, O
miR-197	Down	Plasma	[83]	O
miR-199a	Up	Plasma	[100]	O
miR-20b	Down	Plasma	[83]	A, O
miR-21	Down	Plasma	[83]	O, I, A
miR-222	Up	Plasma	[85]	O, A
miR-223	Down	Plasma	[83]	I, O, A
miR-23a	Down	Serum	[97]	O
miR-24	Down	Plasma	[83]	O, I
miR-28-3p	Up	Plasma	[83]	
miR-29a	Up	Serum	[98]	O
	Up	Peripheral blood	[74]	
miR-29b	Down	Plasma	[83]	O
miR-30d	Up	serum	[98]	O
miR-320a	Down	Plasma	[83]	O
	Up	Peripheral blood	[74]	
miR-326	Up	Plasma	[96]	O
miR-34a	Up	Serum	[98]	O, A, I
miR-375	Up	Serum	[98]	O
miR-423-5p	Down	Plasma	[85]	
miR-486	Down	Serum	[97]	O
	Down	Plasma	[83]	
miR-503	Down	serum	[101]	O, A
	Up	Plasma	[102]	
miR-532-5p	Down	Plasma	[85]	
miR-9	Up	Serum	[98]	O
miR-96	Down	Serum	[97]	O

*CTR*, healthy control subjects; *I*, inflammamiRs; *O*, oncomiRs; *A*, angiomiRs. This classification is based on the amount of relative literature, and some miRNAs can be classified in more than one group

**Table 2:** Cellular miRs differentially expressed in human diabetic and healthy tissue, or in human cell lines exposed to normoglycemic and hyperglycemic conditions

MiRs	Expression levels	mRNA targets	Cell types	Refs	miR classification
Diabetic retinopathy					
miR-146a	Down	Fibronectin	HUVECs	[103]	I, A, O
miR-146b-3p	Down	ADA2	Vitreous of diabetes patients, macrophages	[79]	O
miR-200b	Down	VEGF	HUVEC	[104]	O, A
miR-195	Up	SIRT1	HRECs, HMECs	[105]	A, O
Diabetic nephropathy					
miR-192	Up	ZEB 1/2	Glomeruli from renal biopsies	[106]	O
miR-377	Up	Pak1, Sod1/2	NHMCs	[107]	O, A
miR-29a/b/c	Down	Col1, Col4	h. conditionally immortalized podocyte	[108]	O
miR-21	Up	PTEN, RAS40	hMCs	[91]	O, I, A
	Up	TIMP3	human kidney biopsy	[92]	
miR-155	Up		h. kidney biopsy, HRGECs	[109]	I, O, A
miR-146a	Up		h. kidney biopsy, HRGECs	[109]	I, A, O
miR-215	Down	SIP1/ZEB2	h. conditionally immortalized podocytes	[110]	O
miR-135a	Up	TRPC1	kidney tissues, HMC	[111]	O
Macrovascular diabetic complications					
miR-16	Down	Cox-2	THP-1 monocytes	[112]	O
miR-503	Up	Ccne1-Cdc25A	HUVEC, HMVEC	[102]	O, A
miR-133	Down	Rho-A, Cdc42	h. embryos/fetuses cardiac cells	[113]	I, A
miR-223	Up	Glut4	Left ventricular biopsies	[114]	I, O, A
miR-221	Up	C-kit	HUVEC	[115]	O, A
miR-492	Down	Resistin	HUVEC	[116]	O, A

*H*, human; *HMC*, immortalized human mesangial cell; *HRECs*, *HMECs*, human retinal and dermal microvascular endothelial cells; *HRGECs*, human renal glomerular endothelial cells; *NHMCs*, normal human mesangial cells. *I*, inflammamiRs; *O*, oncomiRs; *A*, angiomiRs. This classification is based on the amount of relative literature, and some miRNAs can be classified in more than one group

#### Future prospects

The continuous interactions between each individual’s genetic makeup and environmental factors result in a spectrum of states that range from healthy aging to age-related impairment and disease. The major age-related diseases, including T2DM, may well be characterized by molecule combinations whose identification would take us a little closer to discovering the biomarkers of health deterioration during aging. Circulating miRNAs, and probably other cell-free nucleic acids, and their shuttles (exosomes and protein/lipoproteins) provide an efficient inter-tissue and inter-organ cross-talk system as well as an integrated reservoir of information relating to all body tissues and organs. The hypothesis that epigenetic modifications may underpin metabolic disorders, including T2DM, and that specific circulating miRNA signatures can have predictive/diagnostic/prognostic relevance in T2DM and related complications is fairly recent. Even though emerging evidence has documented specific circulating miRNA signatures in T2DM, the role of circulating miRNAs in diabetic complications is still largely unexplored. The issue should be investigated in large samples of T2DM patients with and without diabetic complications. Since immune cells and ECs are involved in the most common T2DM complications, their relative contribution to circulating miRNA signatures needs to be elucidated. The investigation of circulating microparticles, including exosomes, in the context of T2DM and its complications is a topical field of inquiry. Exosomes can transport and deliver to target cells not only proteins, but also nucleic acids including miRNAs, DNA, ribosomal RNAs, circular RNAs (circRNAs) and long non-coding RNAs (lnRNAs). Exosomes of endothelial origin could be a source of information on the health status of ECs, serving as reliable systemic biomarkers of ED. Moreover, miRNA-associated exosome administration might be a therapeutic approach to mitigate endothelial activation in T2DM, to avoid or delay the harmful effects of ED-related T2DM complications.

## Conclusions

A range of interventions, including lifestyle modification programs and/or pharmacological treatment, can improve diabetes outcomes. However, these interventions are not sufficient to avoid the onset of the long-term complications of T2DM, and current diabetes parameters are inadequate to predict the likelihood of developing vascular complications by T2DM patients. Therefore, understanding not only genetic variability, but also the mechanisms involved in the interplay of DNA methylation, histone modifications, and miRNAs and their cumulative effect in the context of T2DM and metabolic memory will significantly contribute to the development of novel therapeutic interventions that can delay the harmful effects of the diabetic milieu. Since epigenetic changes are potentially reversible, they are interesting opportunities as targets of new treatments. Combinatorial therapies with conventional drugs and miRNA- or anti-miRNA-treatments are already in progress. MiRNAs targeting methyltransferases or HATs could be used to modulate the epigenetic mechanisms involved in the maintenance of metabolic memory. Moreover, identification of a panel of circulating “epi-markers”, including circulating miRNAs, could revolutionize the management of diabetic patients, enabling the identification of those at increased risk of complications, who require a broader or more aggressive therapy. Many miRNA candidates have already emerged, but further studies are required for their validation in adequate cohorts of T2DM patients with different complications.
